# 

**Published:** 1870-04

**Authors:** 


### CONTENTS.

ORIGINAL COMMUNICATIONS.

Wasted Alveolar Processes and Gums........ 153
Prophylaxis or Prevention of Dental Decay........ 158
Anæsthesia in Surgery.—Who Discovered It?........ 166
A Novel Case and Treatment........ 171
Monument to Dr. Horace Wells........ 172
Not Guilty........ 173

PROCEEDINGS OF SOCIETIES.

Charter of the Kentucky State Dental Association........ 174
New Orleans Dental College........ 175
New Applications to Ulcerated Surfaces........ 176

CORRESPONDENCE.

A Substitute for Vulcanite........ 177
Bases for Artificial Teeth........ 178

SELECTIONS.

Odds and Ends........ 180
Investigations of the Tooth Pulp........ 183
A Shocking Affair.—Death in a Dentist's Office........ 188

EDITORIAL.

"Use Heavy Foils?"........ 191
Failures........ 193
The Southern Dental Association........ 193
Ohio College of Dental Surgery—Commencement Exercises........ 194
Instruments—Pluggers........ 195
Pneumatic Bur Engine........ 195

